# Report on the 40th Annual Meeting of the European Society for Blood and Marrow Transplantation

**DOI:** 10.3332/ecancer.2014.430

**Published:** 2014-05-15

**Authors:** Giuseppe Fatuzzo

**Affiliations:** Humanitas Research Hospital, Via Manzoni 56, 20089 Rozzano, Milan, Italy

**Keywords:** bone marrow transplantation, EBMT, nurses group

## Abstract

**Introduction:**

The EBMT is a non-profit scientific society representing 563 transplant centres from 57 countries in and outside Europe.

**Background:**

From 29 March to 2 April 2014, the EBMT Annual Meeting took place at the Milan Convention Centre (MiCo), for the 40th Annual Meeting.

**Objective/Methods:**

This report will summarise the meeting’s sessions and discuss the nursing scientific programme.

**Data source/extraction:**

programme, interview, presentations, and abstracts.

**Conclusions:**

This year’s 40th Anniversary congress in Milan received a record number of abstract submissions plus an extremely high attendance of more than 4600 participants—the second highest in the history of the EBMT. The meeting provided a venue for a constructive multinational and multidisciplinary dialogue. EBMT continues to monitor differences between countries and spreads good clinical practice.

## EBMT 2014: programme overview

The first images that come to mind when thinking back to the Annual Meeting of the EBMT, which took place between 29 March and 2 April 2014 in Milan, are related to the wide international participation and the wonderful cultural event aimed at the diffusion of a solid and growing body of knowledge of great scientific interest. The EBMT is a non-profit scientific society representing 563 transplant centres from 57 countries [1] in and outside Europe. For 40 years it has significantly contributed to the success of stem-cell transplantation (SCT) through different and various activities addressed to measuring trends in transplant activity, collecting and analysing patient transplant data in Europe, initiating pioneer prospective studies, proving networking opportunities, and educating professionals and establishing strict standards in transplantation procedures, including gathering data in the light of improving patient care.

The 40th edition of the EBMT Annual Meeting took place at the Milan Convention Centre (MiCo), focusing on a multidisciplinary and specific area of expertise in haematological transplantation. This year's event celebrated at the same time the 30th Meeting of the Nurses Group, the 13th Meeting of the Data Management Group, the Sixth Meeting of the Quality Management Group, the Third Paediatric and Cell Therapy Days, the Eighth edition of the Patient & Family Day, and the First Donor Day. This important cultural event fully represented the contemporary vision of a multidisciplinary approach to a topic of interest in health care. Unique of its kind since 1974, it met the growing need for a scientific dialogue between health professionals who seek a point of encounter and collaboration. The recent meeting will be remembered, undoubtedly, for the impressive quality and quantity of accepted abstracts: 187 as oral presentations, 592 as posters, and 281 only as publications. This year’s congress received an impressive attendance of more than 4600 participants, the second highest in the history of the EBMT.

## Nurse Education Day: a designed updated training programme

The fantastic opening section of the ‘Nurse Education Day’, held on 30 March by invitation of expert speakers from the EBMT Nurses Group, was made exceptionally enjoyable by interesting presentations, and by qualified and accredited experts who dealt with different topics focused on the management of early and late effects after haematopoietic stem cell transplantation and their impact on the patient’s quality of life. Experienced nurses from six different nations alternated and presented case studies, the results of literature reviews, the use of validated measurement tools, and the interesting cases of health policies targeting a sophisticated formula to balance cost-effectiveness. A special mention goes to those who closed the proceedings of the day: Sarah J Liptrott for treating brilliantly and comprehensively ‘Body image and self-esteem’ and to Jacqui Stringer for effectively presenting her work on ‘Complementary therapies.’

## Distinguished merit award: interview with Monica Fliedner

Monica Fliedner is well known in the oncology-nursing field; she is currently an advanced nurse practitioner for palliative care at the Berne University Hospital (Switzerland) and has been an active member of the European Blood and Marrow Transplantation Nurses Group (EBMT NG) since she first attended an EBMT conference in 1987. Furthermore, from 2007 until April 2008 she served as president of the EBMT NG.

‘What do you think has been your contribution in more than 20 years at the EBMT?’

She responded with the humility and grace of a confident person who puts you at ease when you first meet her. It is clear that her focused commitment to give a voice to nurses has been and will continue to be her main goal. She confirmed that her contribution in all these years has been to promote and advocate the importance of the voice of the nurse. The voice of those nurses who want to show what they do, the voice of those who want to show scientific arguments that reflect the choice of behaviours and clinical decisions adopted in the light of a rationale which is scientifically shared. The voice of a profession, a discipline, which is the result of a strategy to involve clinical, research, and organisational aspects able to support other health related fields in a context of increasingly multidisciplinary care.

‘Where do you think nursing should go in the next few years?’

She replied stressing the importance and fundamental value of excellent data presentation, arguments, and oral presentations, such as those performed during a conference; words, numbers, data, and tables that we use to express our field of expertise. She reported as an example the existence of courses, more and more often present in a university context, which aim to teach the techniques and provide the necessary knowledge for delivering effective presentations. Different skills are needed to present information correctly and we should invest in them. Development of these communication skills can significantly enhance the level of dialogue among different health care professionals and inevitably raise the level of our profession.

Finally, to the question *‘What are the first three nursing priorities that we should keep in mind in Europe concerning haematological patients' care?’*

She listed with marked decision: patient and family education, management of symptoms, and related nursing interventions, and last but not least, palliative care. She enjoyed this year’s conference, and I can say that she was highly applauded when she was surprised but very delighted to receive the distinguished merit award, for which she thanked the EBMT-NG members for this award of outstanding achievement and felt it was a precious honour.

## Nursing Annual Meeting: an opportunity for insight to master current nursing practice

In the EBMT NG closing session, initial data for the three-day congress were presented. It was attended by 517 nurses from all over the world, the highest nursing attendance ever, with participation at six sessions of oral presentations, various plenary sessions, a nursing symposium, and seven different poster sessions for a total of 80 nursing posters presented and collected in the scientific programme. This year, with great pride, the Scientific Committee announced that 186 nursing abstracts had been submitted, from which 137 were considered for oral presentation or posters—the highest number ever in terms of submissions, following only the EBMT held in Florence in 2008, when 170 were received.

At the meeting held this year, there were 25 submitting countries, of which the UK and Italy had the highest submission rates. If we look back to 1988, the EBMT NG asked its members for the first time to communicate the research projects that were carried out in their units. In 1996, the EBMT NG Research Sub Committee was setup, and nurses reached an important milestone. From that time research was the beginning of a continuous development of evidence-based practice. Now even more than ever, that intellectual curiosity keeps living and great attention has been paid to all the issues raised by the Nurse Group Research Sub Committee about the correct procedures for the submission of a research proposal, the different and common ways of raising funds to support research projects and the huge availability offered by the Committee to support developing activities for setting up new qualitative and quantitative research projects.

The nursing scientific programme was of great interest and included the following areas: paediatric issues, quality of life, disease updates, GVHD, alternative care, palliative care, central venous access devices, symptom management, infection, aphaeresis, patient information and education needs, caregivers, new therapies, complications, late effects, and transplant related issues. Under the guidance of competent chairs these days saw a succession of leading experts discussing the most controversial topics that even with the support of interactive sessions gave rise to debates also including personal experiences and patient case study scenarios.

The Paediatric Committee also gave its remarkable contribution this year. With 143 contact nurses and 22 affiliated countries, it is famous for having developed an active collaboration with the Paediatric Diseases Working Party and performing Master Classes in principals of patient care in both paediatric and adult settings in St Petersburg and Moscow during 2013. It is an example of the successful collaboration between nurses and physician. Also this year, the Committee has shown that it carries on aiming to improve the care of paediatric and adolescent SCT patients and to promote, develop, and share knowledge between paediatric nurses. The participants contributed to the success of this edition with questions, observations, and comments, and, in particular, with visible enthusiasm, which ended with the award of the best poster and best oral presentation abstracts: they went respectively to ‘Evaluation of a discharge booklet for allograft patient’ by Tardieu *et al* [2] with the participation of 37 French centres and ‘Medication non-adherence to taking immunosuppressant's after allogenic hematopoietic stem cell transplantation is associated with cGVHD: PROVIDOMED—a multicentre cross-sectional study’ conducted by Gresch *et al* [3] in Basel, Switzerland.

## Conclusion

From 22 to 25 March 2015 the 41st Annual Meeting of the European Society for Blood Marrow Transplantation will take place in Istanbul, Turkey, which is a terrific cultural bridge between Europe and Asia. Expectations for next year are very high, and this bodes well. It would be superfluous to say more, but it is clear that the EBMT nursing group is proving year after year to provide information, national and international forums to support and share knowledge in research, and education and training by setting up and spreading good clinical practice. This is the formidable result of acknowledging differences between countries and providing a venue for a constructive multinational dialogue.

## Conflict of interest

The author has no conflicts of interest to declare.
